# Healthcare costs for hospitalized COVID-19 patients in a Japanese university hospital: a cross-sectional study

**DOI:** 10.1186/s12962-023-00453-9

**Published:** 2023-07-16

**Authors:** Shunsuke Uno, Rei Goto, Kimiko Honda, Machiko Tokuda, Hirofumi Kamata, Shotaro Chubachi, Ryo Yamamoto, Yukio Sato, Koichiro Homma, Sho Uchida, Ho Namkoong, Yoshifumi Uwamino, Junichi Sasaki, Koichi Fukunaga, Naoki Hasegawa

**Affiliations:** 1 Department of Infectious Diseases, Keio University School of Medicine, 35, Shinanomachi, Shinjuku, Tokyo Japan; 2 Graduate School of Business Administration, Keio University, Yokohama, Kanagawa Japan; 3 Graduate School of Health Management, Keio University, Fujisawa, Kanagawa Japan; 4 Center of Health Economics and Health Technology Assessment, Keio University Global Research Institute, Tokyo, Japan; 5 Division of Pulmonary Medicine, Department of Internal Medicine, Keio University School of Medicine, Tokyo, Japan; 6 Department of Emergency and Critical Care Medicine, Keio University School of Medicine, Tokyo, Japan; 7 Department of Laboratory Medicine, Keio University School of Medicine, Tokyo, Japan

**Keywords:** COVID-19, Economic evaluation, Healthcare costs, Hospitalization, Hospital length of stay

## Abstract

**Background:**

A health-economic evaluation related to COVID-19 is urgently needed to allocate healthcare resources efficiently; however, relevant medical cost data in Japan concerning COVID-19 are scarce.

**Methods:**

This cross-sectional study investigated the healthcare cost for hospitalized COVID-19 patients in 2021 at Keio University Hospital. We calculated the healthcare costs during hospitalization using hospital claims data and investigated the variables significantly related to the healthcare cost with multivariable analysis.

**Results:**

The median healthcare cost per patient for the analyzed 330 patients was Japanese yen (JPY) 1,304,431 (US dollars ~ 11,871) (interquartile range: JPY 968,349–1,954,093), and the median length of stay was 10 days. The median healthcare cost was JPY 798,810 for mild cases; JPY 1,113,680 for moderate I cases; JPY 1,643,909 for moderate II cases; and JPY 6,210,607 for severe cases. Healthcare costs increased by 4.0% for each additional day of hospitalization; 1.26 times for moderate I cases, 1.64 times for moderate II cases, and 1.84 times for severe cases compared to mild cases; and 2.05 times for cases involving ICU stay compared to those not staying in ICU.

**Conclusions:**

We clarified the healthcare cost for hospitalized COVID-19 patients by severity in a Japanese university hospital. These costs contribute as inputs for forthcoming health economic evaluations for strategies for preventing and treating COVID-19.

## Background

The novel coronavirus disease 2019 (COVID-19) has spread rapidly worldwide. Most patients experienced mild disease, while some severe patients needed treatment with intubation and ventilator [1]. Significant effort has been devoted worldwide to the development of various drugs for treating and containing COVID-19; however, COVID-19 remains a social and public health threat.

COVID-19 is highly contagious and can be transmitted even before the onset of the disease [2]; therefore, COVID-19 outbreaks occasionally occur in healthcare facilities [3–5]. Nosocomial infection control measurements for COVID-19 require significant healthcare resources, including human or physical resources, to prevent COVID-19 outbreaks. As a result, the COVID-19 surges in Japan have forced hospitals to limit hospitalization or visits for patients of other diseases [6, 7]; therefore, conducting a health economic evaluation related to COVID-19 is urgently needed to allocate healthcare resources efficiently. Additionally, the result of cost-effectiveness evaluations could also contribute to the improvement of price determination of therapeutic drugs or vaccines. Although all the charges for inpatient treatments against COVID-19 were paid by both the central government through tax and public health insurers in 2021, relevant medical cost data on COVID-19 is scarce in Japan. Japanese claims-data analysis used data from the first wave of the pandemic, reporting an increase in hospital charges from Japanese yen (JPY) 878,352 to JPY 973,584 per overall hospitalized patient [8]; however, how much was consumed for hospitalized COVID-19 patients by severity is unclear.

This study clarifies the total healthcare costs consumed in COVID-19 inpatient care by severity of illness.

## Methods

### Study design and settings

We conducted a cross-sectional study from the public healthcare payer’s perspective to investigate the total healthcare costs (the monetary value of healthcare resources used) for hospitalized COVID-19 patients in Keio University Hospital. Thus, this study did not include the travel and time costs for patients and their family members. Keio University Hospital is a tertiary care hospital in central Tokyo with 946 beds, and has been accepting COVID-19 patients by flexibly increasing the number of beds for COVID-19 patients according to the epidemic status and severity of cases. In reality, Keio University Hospital reserved 25–37 beds for COVID-19 and accepted patients. Patients with up to moderate II COVID-19 were treated by 3–6 internal physicians dedicated to COVID-19 in turns, whereas patients with severe disease were treated by 15–25 intensivists. The general ward and high care units provided care with one nursing staff per four patients, and the ICU provided care with one nursing staff per one patient. Other staff contributed in the same way as for patients admitted for reasons other than COVID-19; therefore, no additional human resources were consumed. We collected the hospital claims data of COVID-19 patients in 2021 and their clinical data from electronic medical records.

The Keio University School of Medicine Ethics Committee approved this study protocol (20221080). Individual informed consent was waived due to the retrospective cross-sectional nature of this study.

### Patients and costs calculation

This study included patients diagnosed with COVID-19 and hospitalized at Keio University Hospital from January 1 to December 31, 2021. The COVID-19 diagnosis was confirmed by a polymerase chain reaction of saliva or nasopharyngeal swab samples. We excluded patients whose disease-causing-hospitalization was not COVID-19 in the Diagnosis Procedure Combination/Per-Diem Payment System (DPC/PDPS) information registered at admission.

DPC/PDPS is a Japanese case-mix system, under which fixed medical payments and additional service fees are determined under public health insurance in Japan. A fee-for-service hospitalization for COVID-19 was partially covered by public health insurance based on the patient’s age, and the remainder was covered by the central government in 2021. Thus, the patients could receive all services without out-of-pocket payments for COVID-19 hospitalization. If complications or underlying diseases were treated, the amount not covered by the public health insurance was paid out-of-pocket. Given the challenges in separating complications related to COVID-19 during hospitalization from those not related to COVID-19, we calculated the total healthcare costs during hospitalization for analyzed patients.

The total healthcare costs were classified into the first visit or revisit, disease management, medication (oral drug and prescription), injection (injectable drug and injection), procedure, surgery, anesthesia, blood transfusion, examination, diagnostic imaging, hospitalization, and others. Temporary charges for in-hospital infection control and additional nurse staffing for COVID-19 [9, 10] were included in the hospitalization fee. The fee for the drugs against COVID-19, such as remdesivir, sotrovimab, and casirivimab/imdevimab were not considered in 2021 because the government procured and provided these drugs free of charge to medical facilities. The calculated total healthcare costs were then multiplied by the rate of change (-0.94% [11]) in reimbursement in April 2022, and the listed price of remdesivir was added for estimating the costs at the end of 2022 to bring the price index as close as possible to the latest value [12].

The hospital claims data also included patient birthdate, sex, date of admission, discharge date, stay required in the intensive care unit (ICU), length of ICU stay, ventilator required, and the length of ventilator use. We calculated the age at admission and hospital days using claims data and collected data from the electronic medical chart on the severity of COVID-19, in-hospital mortality, and the question of whether the patients were transferred from another hospital on the day they were admitted to the hospital. The severity of COVID-19 was used as defined by the Ministry of Health, Labour and Welfare (MHLW), Japan (Table 1 [13–15]). We calculated 1 United States Dollar (USD) as 109.88 JPY at the mean rate in 2021 [16].

**Table 1:** Definition of the severity of COVID-19 by the Ministry of Health, Labour and Welfare

Severity	Definition
Mild	People who have no respiratory symptoms or cough, only; no shortness of breath
Moderate	People who have shortness of breath and pneumonia findings
Moderate I	People who do not need oxygen supply
Moderate II	People who need oxygen supply
Severe	People who need hospitalization to ICU using a ventilator face life-threatening crises

*ICU* intensive care unit

### Statistical analysis

We showed quantitative variables with median and interquartile range (IQR). We estimated the following regression model to investigate the variables significantly related to the total healthcare cost of COVID-19 inpatients.

$${logY=\beta }_{0}+{\beta }_{1}\cdot Age+{\beta }_{2}\cdot Sex+{\beta }_{3}\cdot {d}_{1}+{\beta }_{4}\cdot severity+{\beta }_{5}\cdot ICUstay+{\beta }_{6}\cdot ICUstay\cdot {d}_{2}+{\beta }_{7}\cdot death+\epsilon$$

*Y* is total healthcare cost (JPY); *Age* is the patient’s age at admission; *Sex* is the sex of a patient; ${d}_{1}$ is hospital days; *severity* is the severity of COVID-19, a dummy variable based on the mild patient; *ICUstay* is a dummy variable based on not staying in the ICU during hospitalization; ${d}_{2}$ is the length of ICU stay (days); and *death* is a dummy variable based on the patient’s being discharged alive. *Y* generally had a positively skewed distribution [17]; thus, we performed log transformation, or log link analyses. We performed analyses using generalized linear models assuming normal or gamma distributions and additive or multiplicative error models and compared Akaike’s Information Criteria (AIC); the final model with the lowest AIC was adopted for model specification. The variance inflation factor (VIF) was calculated to confirm the absence of multicollinearity. We calculated the multiplier of change in healthcare cost per unit of the independent variable by calculating ${e}^{\beta }$.

All statistical analyses were performed using R ver. 4.1.2 (The R Foundation for Statistical Computing, Vienna, Austria).

## Results

**Fig. 1:**
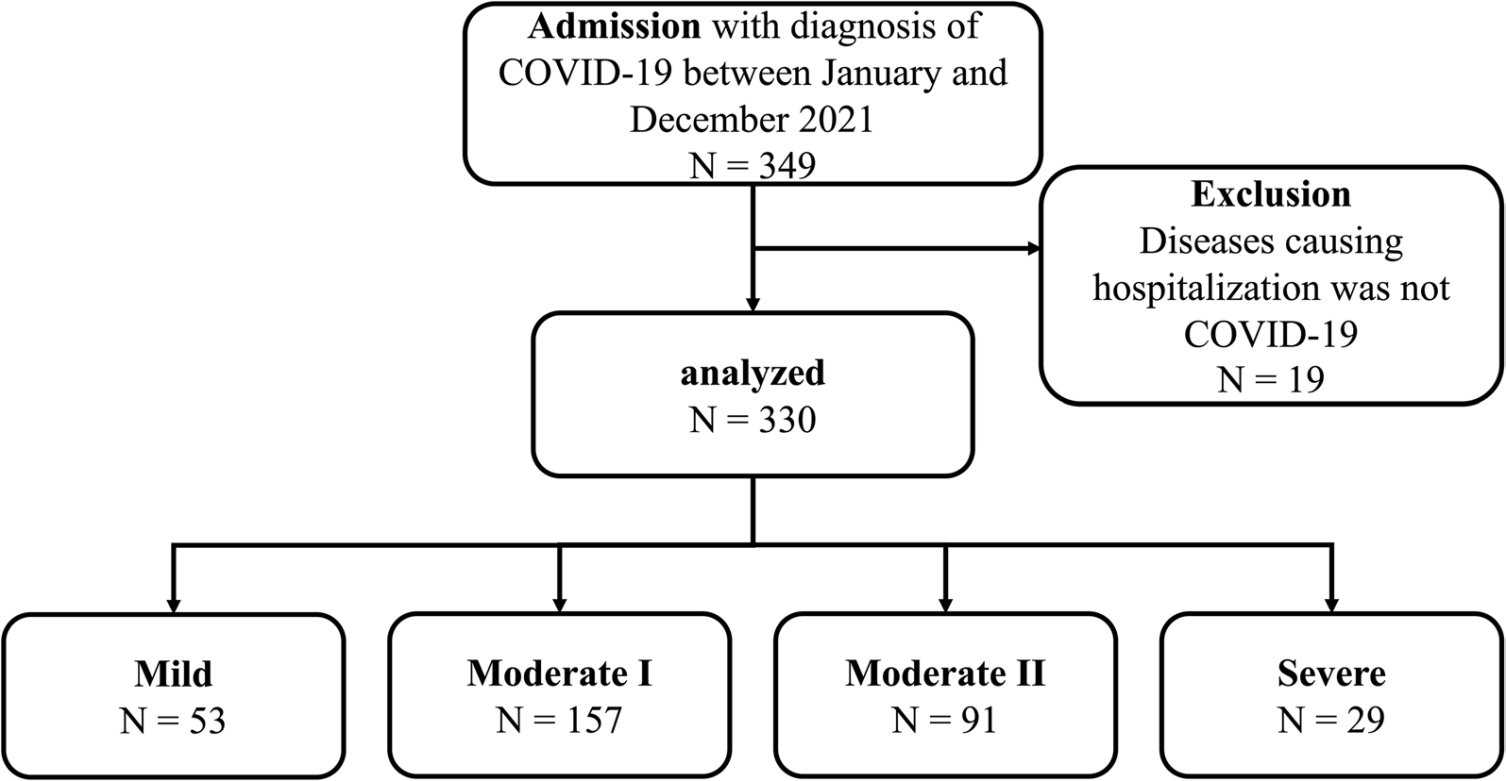
Study participants

During the study period, 349 hospitalized COVID-19 patients were identified, and 330 were analyzed after excluding 19 patients (Fig. 1). Table 2 presents the patient characteristics. Older and male patients tended to be more prevalent in the severe group. The median hospital days was 10 (IQR: 8–14) and the more severe group tended to have longer hospital days. The moderate II group included nine patients who initially admitted to the ICU because of concerns of their conditions deteriorating; however, their COVID-19 severity did not deteriorate and clinically determined to be moderate II. The in-hospital mortality rate was 0% for mild and moderate I, 2.2% (2/91) for moderate II, and 17.2% (5/29) for the severe group.

**Table 2:** Patient characteristics

	Total N = 330	Mild N = 53	Moderate I N = 157	Moderate II N = 91	Severe N = 29
Age, median (IQR)	53 (40–62)	43 (30–55)	52 (41–61)	54 (48–63.5)	57 (47–64)
Sex, male (%)	229 (69.4)	32 (60.4)	110 (70.1)	64 (70.3)	23 (79.3)
Hospital days, median (IQR)	10 (8–14)	8 (7–10)	9 (7–11)	12 (10–15)	17 (14–26)
ICU stay, n (%)	38 (11.5)	0	0	9 (9.9)	29 (100)
Days of ICU stay, median (IQR)	7 (3–13)	0	0	3 (2–3)	9 (5–16)
Ventilator use, n (%)	20 (6.1)	0	0	0	20 (69.0)
Days of ventilator use, median (IQR)	10 (7–20)	0	0	0	10 (7–20)
Transfer from another hospital at admission, n (%)	25 (7.6)	0	3 (1.9)	4 (4.4)	18 (62.1)
Treated with remdesivir, n (%)	227 (68.8)	5 (9.4)	112 (71.3)	84 (92.3)	26 (89.7)
In-hospital death, n (%)	7 (2.1)	0	0	2 (2.2)	5 (17.2)

*IQR* interquartile range, *ICU* intensive care unit

**Fig. 2:**
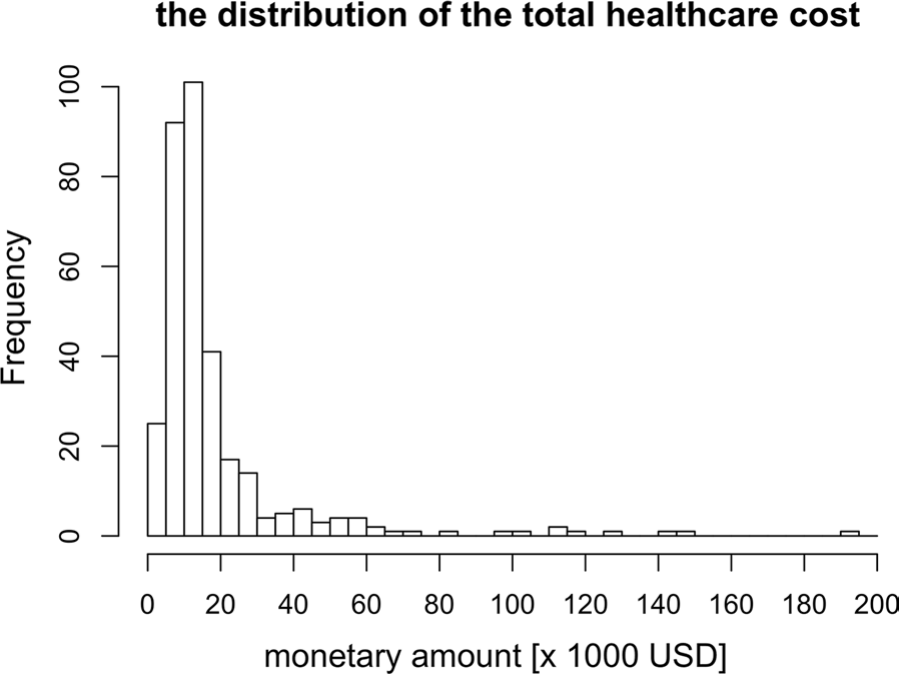
Distribution of the total healthcare cost of hospitalized COVID-19 patients

The estimated total healthcare cost for the analyzed 330 patients hospitalized in 2021 was JPY 678,679,460 (about USD 6,176,551), and the median healthcare costs per patient for hospitalized COVID-19 was JPY 1,304,431 (about USD 11,871) (IQR: JPY 968,349–1,954,093). Figure 2 shows the distribution of the estimated healthcare cost. Table 3 shows the estimated healthcare cost broken down by severity. The median healthcare cost per patient was JPY 798,810 (USD 7270) in mild cases; JPY 1,113,680 (USD 10,135) in moderate I cases; JPY 1,643,909 (USD 14,961) in moderate II; and JPY 6,210,607 (USD 56,522) in severe cases. In moderate II, the median healthcare cost and hospital days were JPY 4,289,207 (USD 39,035) and 15 days, respectively, for those who stayed in the ICU and JPY 1,625,404 (USD 14,792) and 12 days, respectively, for those who did not stay in the ICU, which differed significantly. Hospitalization fees accounted for a large proportion of total healthcare costs. These include the daily inpatient charge, an additional charge for the use of the intensive care unit, for nursing systems coste, and costs for managing the use of ventilators.

**Table 3:** Healthcare cost for hospitalized COVID-19 patients, median (IQR), USD

	Total N = 330	Mild N = 53	Moderate I N = 157	Moderate II N = 91	Severe N = 29
Median healthcare cost	11871.41 (8812.79–17783.88)	7269.84 (5325.78–11622.60)	10135.42 (8324.97–13235.24)	14960.95 (12305.45–22870.28)	56521.72 (43283.16–102245.59)
First visit/revisit*	25.96 (0–31.83)	0 (0–26.41)	25.96 (0–33.63)	26.41 (0–46.70)	26.41 (0–26.41)
Diseases management	49.58 (27.05–56.34)	27.05 (27.05–56.34)	27.05 (27.05–56.34)	54.54 (27.05–58.83)	153.71 (73.03–203.29)
Medication	30.02 (13.21–97.02)	21.37 (7.48–50.04)	21.72 (10.09–49.67)	49.95 (21.23–134.15)	264.23 (97.45–507.20)
Injection	3505.50 (58.57–3882.18)	0.90 (0–21.81)	3494.68 (103.86–3534.79)	3604.84 (3535.78–5366.78)	7462.40 (4894.84–8838.17)
Procedure	0 (0–8.25)	0 (0–0)	0 (0–0)	5.14 (0.18–27.77)	772.97 (241.70–2090.64)
Surgery	0 (0–0)	0 (0–0)	0 (0–0)	0 (0–0)	0 (0–320.50)
Anesthesia	0 (0–0)	0 (0–0)	0 (0–0)	0 (0–0)	0 (0–864.48)
Blood transfusion	0 (0–0)	0 (0–0)	0 (0–0)	0 (0–0)	0 (0–0)
Examination	489.71 (251.35–683.72)	359.53 (251.35–637.56)	494.76 (354.48–680.47)	489.71 (267.57–739.84)	489.71 (373.05–976.54)
Diagnostic imaging	236.20 (208.25–311.49)	198.33 (179.40–227.18)	223.31 (198.33–246.11)	281.28 (239.81–419.53)	685.43 (513.69–1242.04)
Others, rehabilitation, etc.	0 (0–0)	0 (0–0)	0 (0–0)	0 (0–0)	0 (0–63.11)
Hospitalization	7575.73 (5489.77–13483.69)	6179.26 (4389.01–9204.79)	6164.39 (4825.07–8186.42)	10067.91 (7678.28–16106.44)	45265.68 (33809.59–84831.53)

* A first visit fee and an additional fee based on a patient’s age or visiting time to the hospital can be charged as the cost of hospitalization on the first day. As the revisit fee cannot be charged, it is zero for revisit fee

Table 4 presents the result of the regression analysis. A generalized linear model at the multiplicative error model, log-transformed and assuming a gamma distribution yielded the smallest AIC, 365.37. AIC was 9751.5 in the additive error model, and assuming a gamma distribution, it was 367.46 in the multiplicative error model (log-transformed and assuming a gaussian distribution), and 10,176 in the additive error model assuming a gaussian distribution. We interpreted it as no multicollinearity as the highest VIF was 2.06 among all the analyzed variables. We also examined the inclusion of ventilator use and the length of ventilator use as independent variables in the regression analysis model; however, VIF exceeded 5, resulting in suspected multicollinearity, so we excluded those variables. In the analysis, healthcare costs increased by 4.0% for each additional day of hospitalization; 1.26 times for moderate I cases, 1.64 times for moderate II cases, and 1.84 times for severe cases compared to mild cases; and 2.05 times for cases involving ICU stay compared to those not staying in ICU.

**Table 4:** Result of the regression analysis

	β coefficient	Exponentiated β	95% CI of exponentiated β	P value	VIF
(Intercept)	13.3	6.11 × 10^5^	5.09 × 10^5^–7.33 × 10^5^	< 0.05	
Age	5.19 × 10^− 4^	1.001	0.998–1.003	0.72	1.09
Sex					1.01
Male	(ref.)			−	
Female	−0.0312	0.969	0.879–1.069	0.53	
Hospital days	0.0388	1.040	1.031–1.049	< 0.05*	1.27
Severity					1.34
Mild	(ref)			−	
Moderate I	0.228	1.26	1.105–1.426	< 0.05*	
Moderate II	0.497	1.64	1.417–1.908	< 0.05*	
Severe	0.611	1.84	1.235–2.739	< 0.05*	
ICU stay	0.718	2.05	1.501–2.816	< 0.05*	2.06
In-hospital death	0.101	1.11	0.732–1.685	0.63	1.21
Interaction between ICU stay and days of ICU stay	0.0190	1.02	0.995–1.044	0.11	1.95

Akaike’s information criteria: 365.37

*CI* confidence interval, *VIF* variance inflation factor

*Statistically significant

## Discussion

We showed that the total healthcare cost for the analyzed 330 patients hospitalized in 2021 was JPY 678,679,460 (about USD 6,176,551), and the median healthcare costs per patient for hospitalized COVID-19 was JPY 1,304,431 (about USD 11,871) for 10 days of hospitalization duration, most of which were hospital charges. The median healthcare cost per patient was JPY 798,810 (USD 7,270) in mild cases; JPY 1,113,680 (USD 10,135) in moderate I cases; JPY 1,643,909 (USD 14,961) in moderate II cases; and JPY 6,210,607 (USD 56,522) in severe cases. The regression analysis showed that healthcare costs increased by 4.0% for each additional day of hospitalization: 1.26 times for moderate I cases; 1.64 times for moderate II cases; 1.84 times for severe cases compared to mild cases; and 2.05 times for cases involving ICU stay compared to those not staying in ICU. As hospitalization fees accounted for a large proportion of total healthcare costs, it has been speculated that healthcare costs were driven by charges for longer hospital stays, the use of high care units, the additional use of ventilators and the use of more extensive care systems for more severe patients.

The healthcare cost in Japan for hospitalized COVID-19 patients was much higher than that for community-acquired pneumonia, reported as USD 12,728 in ICU episodes and USD 4,824 in general ward episodes between June 2014 and May 2015 [18]. Part of the reason for this discrepancy was that the MHLW first raised temporary payments for emergency room and ICU for COVID-19 patients on April 18, 2020 [9]. They initially doubled the emergency room and ICU fees when additional nurses were assigned to the ward. The MHLW also extended the maximum number of days that could be claimed for ICU stay management fees based on the patient’s condition, from 14 days to 21 or 35 days [9]. In addition, these payments tripled on May 26, 2020 [10]. This was interpreted as incentivizing hospitals to increase the number of beds available for COVID-19 administration or to allow hospitals to use their human resources for infection control and COVID-19 care. That is to say, inpatient care for COVID-19 was implied as requiring medical or human resources than inpatient care for community-acquired pneumonia. Moreover, for a temporary increase in reimbursement to act as an incentive, the reimbursement must be higher than the amount actually spent by the hospital for services. Whether the amount set in Japan this time was appropriate or not will need to be elucidated in future research.

In our results, the median length of hospital stay (LOS) was 10 days, and COVID-19 patients with mild severity were also hospitalized for 10 days (IQR 8–14 days) because the government set the criteria for discharge as 10 days after the onset of illness and 72 h after the resolution of symptoms at that time [19]. In addition, the government’s temporal directive to hospitalize all patients confirmed infected with the variant strain [20] was considered to have contributed to the increased healthcare costs.

The previously reported median healthcare cost per hospitalized patient for COVID-19 among high-income countries was EUR 4435 (about USD 5067 on the average rate in the study period, 2020), and the median LOS was 10 days in Portugal [21], and the corresponding numbers in the US were USD 12,046 and 5 days [22]. As for severe cases, that was EUR 27,748 (about USD 31,702) in the patients who stayed in ICU, and the median LOS was 21 days in Portugal [21], and the corresponding numbers in the US were USD 54,402 in the patients who stayed in ICU and used mechanical ventilation and 15 days [22]. Although the healthcare costs for COVID-19 inpatients varied by country, the healthcare costs in this study were comparable. This finding could indicate that COVID-19 inpatient care similarly requires medical and human resources among countries with similar income levels.

Our result faces some limitations. First, we calculated the monetary value of used healthcare resources on the basis of the claims data during hospitalization at Keio University Hospital. It is important to note that the estimated hospitalization cost was the amount paid but not the actual cost incurred by the hospital to provide services. Furthermore, we did not include the number of healthcare resources used at the previous hospital in cases where the patient was transferred at the time of admission or the amount of rehabilitation after discharge from Keio University Hospital. Therefore, the monetary value of healthcare resources consumed by one patient may be underestimated. Second, A large registry study in Japan through March 31, 2022, with 56.9% men and a median age of 57 years [23], may differ from the population in this study. Although caution should be exercised regarding the generalizability of our result as it was based on data from a single institution, it is presumed that differences in treatment decisions toward COVID-19 by the institution were small in 2021 because there were small treatment options, and treatment guidelines were developed early on. Third, we did not include government subsidies to hospitals or government financial aid to reserve some beds for possible increases in emergent admission of COVID-19 patients. Furthermore, the cost of drugs for COVID-19, such as sotrovimab, and casirivimab/imdevimab, was not included because they are not listed in the Japanese medical fee schedule; thus, this analysis may underestimate the total healthcare costs. Fourth, our analysis did not include costs other than direct medical costs. Direct nonmedical costs (such as travel costs of patients and their families) and indirect costs (such as lost income during hospitalization and time costs of unpaid family care) are not included in this study; the complete healthcare economic burden of hospitalized patients would increase if these were included. Finally, as treatment options, healthcare delivery systems, or legislation for COVID-19 are changing rapidly, the results of the analysis in 2022 and 2023 may differ from that in 2021. Changes from year to year should be elucidated in future research.

## Conclusion

We clarified the healthcare cost for hospitalized COVID-19 patients by severity in a Japanese university hospital. Our data represent inputs for forthcoming health economic evaluation for the prevention and treatment strategy of COVID-19.

## Data Availability

The datasets used and analyzed during the current study are available from the corresponding author upon reasonable request.

## Abbreviations

AIC: Akaike’s information criteria
COVID-19: The novel coronavirus disease 2019
DPC/PDPS: Diagnosis Procedure Combination/Per-Diem Payment System
ICU: Intensive care unit
IQR: Interquartile range
JPY: Japanese yen
MHLW: The Ministry of Health, Labour and Welfare
USD: United States dollars
VIF: Variance inflation factor
